# A Simplified and Robust Model for the Study of Diabetic Nephropathy: Streptozotocin-Induced Diabetic Mice Fed a High-Protein Diet

**DOI:** 10.3390/ijms26062477

**Published:** 2025-03-10

**Authors:** Océane Pointeau, Romain Barbosa, Maéva Loriot, Julia Leemput, Elisabeth Dubus, Sébastien Zwe Causse, Laurent Demizieux, Patricia Passilly-Degrace, Pascal Degrace, Bruno Vergès, Tony Jourdan

**Affiliations:** 1 UFR Sciences Vie Terre Environnement, Université Bourgogne Europe, 21000 Dijon, France; 2 INSERM Research Center U1231, Center for Translational and Molecular Medicine (CTM), Team Pathophysiology of Dyslipidemia (PADYS), 21000 Dijon, France; 3 ImaFlow Core Facility, US58 BioSanD, INSERM, Université Bourgogne Europe, 21000 Dijon, France; 4 Department of Endocrinology, Diabetology and Metabolic Diseases, University Hospital, 21000 Dijon, France

**Keywords:** C57Bl/6J mice, high protein diet, streptozotocin, diabetic nephropathy

## Abstract

To better understand diabetic nephropathy (DN), developing accurate animal models is crucial. Current models often fail to fully mimic human DN, showing only mild albuminuria, glomerular hypertrophy, and limited mesangial matrix expansion. Our study aims to develop a more robust model by combining streptozotocin (STZ)-induced diabetes with a high-protein diet (HPD). We divided C57Bl/6J mice into three groups: control, STZ with a standard diet (STZ-SD), and STZ with a HPD (45 kcal% protein) (STZ-HPD) for 12 weeks. Renal function was evaluated using the urinary albumin-to-creatinine ratio, and kidney tissues were analyzed for histological and molecular changes. The STZ-HPD group showed significantly higher albuminuria and more severe glomerular and tubular damage compared to the control and STZ-SD groups. These changes were accompanied by increased inflammatory and oxidative stress markers, highlighting the harmful effects of high-protein intake on renal injury. Our findings suggest that the STZ-HPD model could be a valuable tool for studying DN pathophysiology and evaluating therapeutic interventions, providing a new approach for preclinical research.

## 1. Introduction

Diabetic nephropathy (DN) is a severe microvascular complication of diabetes, characterized by progressive damage to the kidneys. It is the leading cause of end-stage renal disease worldwide, with a significant impact on morbidity and mortality [1,2]. The prevalence of DN varies widely depending on the population and the diagnostic criteria used. In the United States, it is estimated that approximately 30–40% of individuals with diabetes will develop DN [1,2]. The prevalence is higher in certain populations, such as Native Americans, African Americans, and Hispanics/Latinos. The etiology of DN is complex and multifactorial, involving both hemodynamic and metabolic factors. Hyperglycemia is a key driver of the disease, leading to the accumulation of advanced glycation end-products (AGEs), oxidative stress, and inflammation. These processes result in the thickening of the glomerular basement membrane, the accumulation of extracellular matrix proteins, and the eventual loss of kidney function [3,4].

Several studies have documented that dietary protein intake can modulate the course of chronic kidney disease (CKD), and that protein restriction exerts a beneficial effect on disease progression [5,6,7,8,9,10,11]. Indeed, high-protein diets can potentially exacerbate existing kidney damage in individuals with diabetic nephropathy by increasing the workload of the kidneys [5]. The Brenner hypothesis states that high-protein diets can lead to increased glomerular pressure, which in turn increases the glomerular filtration rate (GFR). This is thought to be due to the vasodilatory effects of certain amino acids, such as arginine and glycine, which can dilate the afferent arteriole, the blood vessel leading into the glomerulus [5]. Moreover, it is now well demonstrated that these diets can stimulate the renin–angiotensin system, which in turn induces the vasoconstriction of the efferent arteriole of the glomeruli [12,13]. Both vasodilation of the afferent arteriole and vasoconstriction of the efferent arteriole lead to increased glomerular pressure and, thus, to alleviate its structure and function. In addition, high-protein intake is associated with increased production of nitrogenous wastes such as urea, which further leads to an increase in GFR [14].

Similar to humans, protein intake has a significant effect on the development of nephropathy in diabetic mice [15] and a high protein intake in db/db mice can significantly increase both albuminuria and histological renal remodeling [16]. Recently, Nørgaard and collaborators have published an elegant study using high-protein diet-fed C57BLKS-Lepr^db/db^ mice demonstrating that these mice displayed a significant increase in several readouts of renal injury, including albuminuria, renal hypertrophy, mesangial expansion, as well as collagen III deposits after 9 weeks on the diet [17]. Renal fibrosis is also impacted by high-protein intake, as several studies highlight dysregulated production of pro- and anti-fibrotic growth factors and cytokines such as TGF-β, endothelin 1, TNF-α, IL-6, and BMP-7 [18,19].

Thus, the development and use of animal models play a critical role in better understanding the etiology and pathogenesis of DN. However, no single model fully recapitulates human pathology, as most of them only manifest modest albuminuria, glomerular hypertrophy, and little mesangial matrix expansion [20,21]. Here, we describe an easy-to-implement model we developed by feeding a high-protein diet to streptozotocin (STZ)-induced diabetic C57Bl/6J mice for 12 weeks (STZ-HPD). Compared to the usual STZ model, which is known to induce an increase in the urinary albumin-to-creatinine ratio, glomerular and tubular hyperplasia, as well as tubular cell injuries associated with inflammation, protein intake further amplifies these alterations and enhances the inflammatory response. As such, this model appears suitable for the study of the pathophysiology of DN.

## 2. Results

### 2.1. Impact of Treatments on Diabetes-Related Parameters

**Figure 1:**
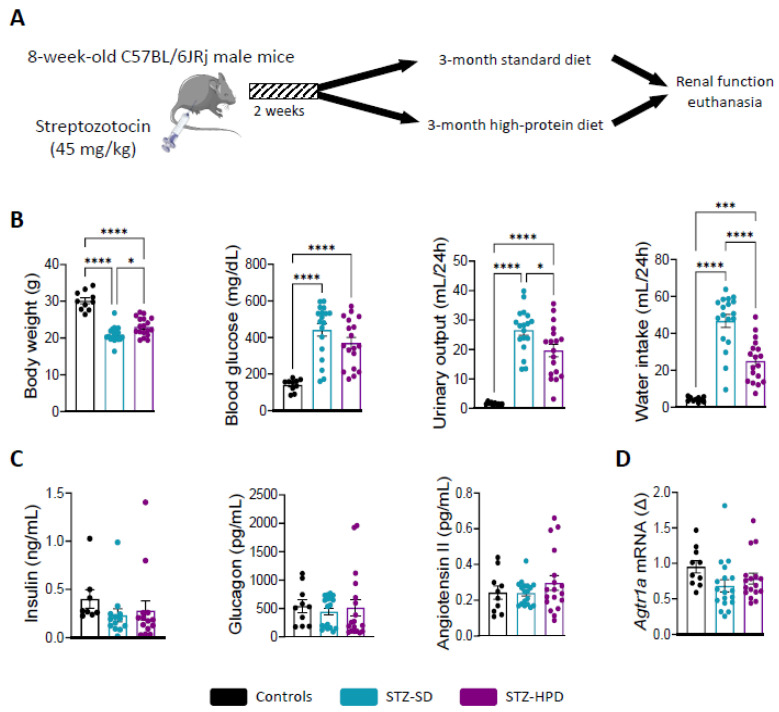
Impact of treatments on diabetes-related parameters. (**A**) *In vivo* experimental outline. (**B**) Body weight, glycemia, polyuria, and water intake. (**C**) Circulating levels of Insulin, glucagon, and angiotensin II. (**D**) *Agtr1a* mRNA expression in renal cortices. Black dots represent the control mice (*n* = 10), the light blue dots represent the STZ-SD mice (*n* = 18), and the purple dots represent STZ-HPD mice (*n* = 18). Data represent mean ± SEM. Statistical significance: * *p* < 0.05; *** *p* < 0.001; **** *p* < 0.0001.

The current study involved 46 mice derived from two independently conducted, yet identically executed experiments. Of these, 10 mice were randomly designated as non-diabetic controls (receiving injections of citrate buffer), whereas the other 36 mice were administered STZ to induce diabetes. Following a 2-week post-injection period, all STZ-treated mice demonstrated blood glucose levels of ≥230 mg/dL and were randomly distributed into one of two dietary conditions: a standard diet (STZ-SD) or a high-protein diet (STZ-HPD) (Figure 1A).

After a 3-month dietary intervention, diabetic mice exhibited comparable weight loss and hyperglycemia to control mice, regardless of the diet (Figure 1B). These findings suggest that any difference observed regarding renal physiology and functionality between the two groups is not glucose-dependent. Compared to control mice, both STZ-SD and STZ-HPD mice showed increased urinary output and water intake (Figure 1B).

Interestingly, HPD lowered the magnitudes of these parameters. Three months after diabetes induction, mice exhibited slightly lower insulin levels (*p* < 0.4458) compared to controls, with no diet effect observed (Figure 1C). STZ is known to selectively target and induce pancreatic beta cell death, while alpha cells remain intact. This non-significant result may reflect partial beta cell destruction from STZ injection or beta cell regeneration over the 3-month dietary period. Additionally, we did not observe any differences in the levels of circulating glucagon and angiotensin II, along with angiotensin II receptor (*Agtr1a*) gene expression, in renal cortices between the three groups (Figure 1C,D).

### 2.2. Impact of High-Protein Diet on Renal Glomeruli

**Figure 2:**
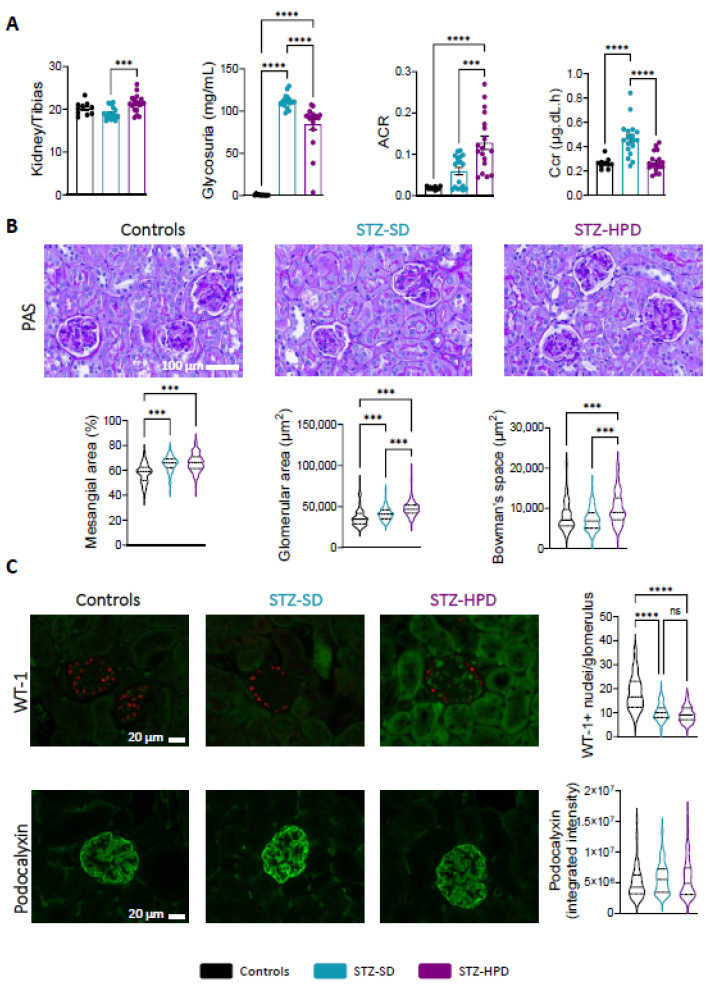
Effects of dietary intervention on renal glomeruli. (**A**) Relative kidney mass, glycosuria, urinary albumin-to-creatinine ratio (ACR), and creatinine clearance (Ccr). (**B**) Representative Periodic Acid Schiff (PAS) staining performed on histological renal sections (scale: 100 µm). Mean mesangial area, glomerular area, and Bowman space quantification. (**C**) First row: Representative immunostaining for Wilms tumor-1 protein (WT-1) in red and auto-florescence in green (scale: 20 µm), with mean podocyte numbers per glomerulus. Second row: Representative immunostaining for podocalyxin protein (scale: 20 µm) with its signal quantification. Black dots represent control mice (*n* = 10), the light blue dots represent STZ-SD mice (*n* = 18), and the purple dots represent STZ-HPD mice (*n* = 18). Data represent mean ± SEM. Statistical significance: *** *p* < 0.001; **** *p* < 0.0001; ns = not significant.

STZ-HPD mice displayed a noticeable increase in kidney weight compared to STZ-SD mice (Figure 2A), likely due to renal hypertrophy. Additionally, glycosuria was elevated in both STZ-SD and STZ-HPD mice, with a lower extent in the latter (Figure 2A). The albumin-to-creatinine ratio (ACR) tended to increase in STZ-SD mice compared to controls (*p* = 0.107), while this parameter was strongly elevated in STZ-HPD mice (Figure 2A), indicating increased albuminuria and glomerular damage, thus reflecting a worsened renal function. Furthermore, creatinine clearance (Ccr) was increased in STZ-SD mice compared to controls, suggesting that these animals are in a hyperfiltration state, while STZ-HPD mice displayed Ccr comparable to controls (Figure 2A).

Collectively, these findings imply that a high-protein diet exacerbates renal dysfunction under diabetic conditions, as evidenced by kidney hypertrophy, decreased glycosuria, and increased albuminuria. In DN, these renal dysfunctions are associated with glomerulopathy characterized by an increase in glomeruli size and mesangial space. As expected, STZ-SD mice displayed a higher mesangial and glomerular surface area compared to control mice (Figure 2B). Interestingly, we found that a higher protein intake led to a stronger increase in glomerular surface area and Bowman’s space, indicating a larger glomerular hypertrophy (Figure 2B). Glomerular dysfunction is frequently linked to podocyte loss. Accordingly, we quantified podocyte levels by assessing the number of Wilms tumor 1 (WT-1)-positive nuclei per glomerulus. Compared to control mice, we observed a decrease in podocytes numbers in both STZ-SD and STZ-HPD mice, with no additional effect of higher protein intake (Figure 2C). Then, we evaluated the expression levels of podocalyxin to determine their integrity and their functionality. We found no changes in podocalyxin intensity across the three groups (Figure 2C). Altogether, these data suggest that higher protein intake might lead to milder increase in diabetes-induced glomerular dysfunction.

### 2.3. Impact of High-Protein Diet on Renal Tubules

**Figure 3:**
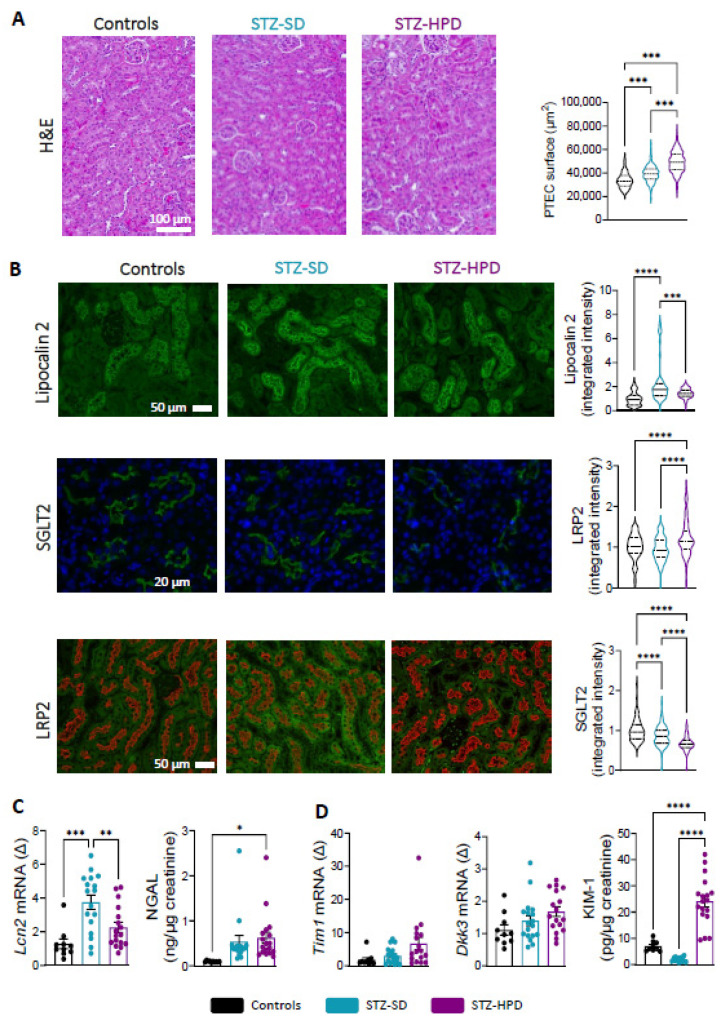
Effects of dietary intervention on renal tubules. (**A**) Representative H&E staining of renal slides (scale: 100 µm) with PTEC mean surface area analysis. (**B**) Representative immunostaining and signal quantification for Lipocalin-2 protein (scale: 50 µm), SGLT-2 protein (green, nuclei in blue; scale: 20 µm), and megalin protein (LRP2) in red and auto-florescence in green (scale: 50 µm). (**C**) *Lcn2* mRNA expression in renal cortices and Neutrophil Gelatinase-Associated Lipocalin (NGAL) urinary excretion. (**D**) *Dkk3* and *Tim1* mRNA expression in renal cortices and KIM-1 urinary concentration. Black dots represent control mice (*n* = 10), light blue dots represent STZ-SD mice (*n* = 18), and purple dots represent STZ-HPD mice (*n* = 18). Data represent mean ± SEM. Statistical significance: * *p* < 0.05; ** *p* < 0.01; *** *p* < 0.001; **** *p* < 0.0001.

DN is frequently linked to tubulopathy, which is in part characterized by proximal tubular epithelial cell (PTEC) lesions and hyperplasia. Compared to controls, STZ-SD mice displayed a significant increase in PTEC surface area indicating hyperplasia typical of DN (Figure 3A). This parameter was further increased in the STZ-HPD group, suggesting that elevated protein intake amplified metabolic stress on renal tubules, potentially accelerating nephropathy progression (Figure 3A). Over the course of DN, damage to PTEC usually induces an increase in the expression of the glycoprotein Lipocalin-2 [22]. As expected, Lipocalin-2 was elevated in the STZ-SD group but, surprisingly, not in the STZ-HPD group (Figure 3B). A similar pattern was observed for Lipocalin-2 (*Lcn2*) gene expression, whereas we found an increase in its urinary excretion (NGAL) in the STZ-HPD group only when compared with control mice (Figure 3C).

We next investigated the impact of diabetes and protein intake on the expression of sodium–glucose cotransporter 2 (SGLT2). We found a reduction in SGLT2 in both STZ-SD and STZ-HPD mice, with a more pronounced decrease in the latter (Figure 3B). Concerning megalin (LRP2), which plays a role in both protein reabsorption and detoxification processes, its staining intensity was only increased in the STZ-HPD group (Figure 3B).

Finally, we analyzed other markers of tubular injury, namely Dickkopf WNT Signaling Pathway Inhibitor 3 (DKK3) and Kidney Injury Molecule 1 (KIM-1). We did not observe any statistical differences in *Dkk3* and *Tim-1* (encoding for KIM-1) gene expression between groups (Figure 3D), although levels were higher in STZ-HPD mice (*p* = 0.067 for *Dkk3* and 0.051 for *Tim-1* compared to controls). Interestingly, urinary excretion of KIM-1 was strongly increased in the STZ-HPD group (Figure 3D). Overall, these results show that the high-protein diet appears to intensify renal tubulopathy, potentially accelerating nephropathy progression.

### 2.4. Impact of High-Protein Diet on Renal Fibrosis

**Figure 4:**
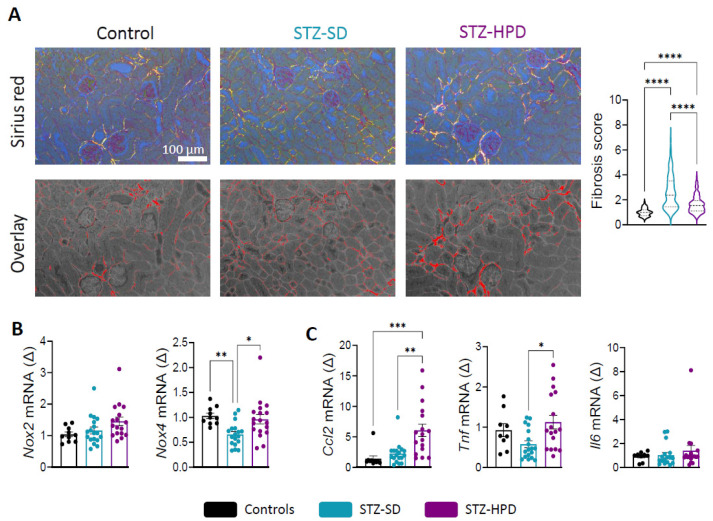
Effects of dietary intervention on tubulo-interstitial fibrosis. (**A**) Representative Sirius red stainings of renal sections (scale: 100 µm) with fibrosis score analysis. (**B**) *Nox2* and *Nox4* mRNA expression. (**C**) *Ccl2*, *Tnf,* and *Il6* mRNA expression. Black dots represent the control mice (*n* = 10), the light blue dots represent the STZ-SD mice (*n* = 18), and the purple dots represent STZ-HPD mice (*n* = 18). Data represent mean ± SEM. Statistical significance: * *p* < 0.05; ** *p* < 0.01; *** *p* < 0.001; **** *p* < 0.0001.

Analysis of fibrosis by Sirius Red staining showed a marked increase in fibrosis scores in both STZ-SD and STZ-HPD mice compared with controls, indicating significant renal damage characteristic of DN (Figure 4A). Surprisingly, the STZ-HPD group displayed a slightly lower fibrosis score than the STZ-SD mice (Figure 4A). This suggests that, although a high-protein diet may worsen some renal cellular damage, at both glomerular and tubular level, it may also limit the extent of fibrosis via mechanisms that remain to be determined.

Oxidative stress and inflammation are key contributors to renal damage and fibrosis. To explore these aspects, we analyzed the expression of two markers of oxidative stress, NADPH oxidase 2 and 4 (*Nox2* and *Nox4*), along with inflammatory markers such as *Ccl2*, *Tnf,* and *Il6*. Our results revealed no significant changes in *Nox2* expression across groups, although it was slightly increased in the STZ-HPD group compared to controls (*p* = 0.075) (Figure 4B). Regarding *Nox4* expression, we observed a decrease in the renal cortex of the STZ-SD group compared to controls, while high protein intake prevented this effect in the STZ-HPD group (Figure 4B).

Regarding inflammatory markers, *Ccl2* expression was strongly increased in STZ-HPD mice compared to both controls and STZ-SD mice (Figure 4C). As *Ccl2* is a crucial chemokine responsible for recruiting monocytes to sites of inflammation, this elevation may indicate an enhanced inflammatory response driven by the additional protein intake, potentially worsening the renal inflammatory environment associated with DN. Interestingly, STZ-HPD mice displayed stronger *Tnf* expression than the STZ-SD group, while no changes were observed regarding *Il6* expression in the three groups (Figure 4C).

Overall, these findings indicate that the high-protein diet appears to amplify oxidative and inflammatory stress in diabetic mice, which may contribute to a progression of DN.

## 3. Discussion

In this study, we provide evidence that the STZ-induced diabetic mouse model coupled with a high-protein diet represents a novel and emerging approach to studying DN, offering significant advantages over both the traditional STZ model and the db/db mouse model. This combined model effectively amplifies the renal-specific pathophysiological features of DN by adding a dietary metabolic stressor, making it a more robust and translatable system for DN research. Indeed, STZ predominantly induces hyperglycemia and early-stage DN, while the addition of a high-protein diet accelerates and exacerbates key DN hallmarks, including tubular hypertrophy, oxidative stress, and inflammation.

The literature on db/db mice has mostly focused on the improvement of renal manifestation by low protein intake [15,23]. However, protein intake has been documented to exert significant deleterious effects on the development of nephropathy in diabetic mice [15,16,23,24]. Specifically, a study published in 1987 showed that high protein intake by db/db mice can significantly increase both albuminuria and histological renal changes [16]. It is also noteworthy to point out that a low-protein diet improves the conservative management of non-dialysis-dependent CKD and could be a viable option for CKD patients aiming to delay or avoid dialysis and slow the progression of their condition [10,11].

In addition to presenting a budgetary challenge, db/db mice also have the major disadvantage of displaying impaired leptin signaling. Indeed, leptin has both direct and indirect deleterious effects on renal function [25] as it directly participates in the development of the thickening of the basement membrane of the proximal tubular cells (which increases protein leakage into filtrate) and the activation of protein synthesis [26,27]. By inducing the production of proinflammatory mediators [28] and reactive oxygen species [29], leptin may also contribute to the inflammatory process found in renal disease and, thereby, cardiovascular complications. In this regard, using a wild-type animal appears to be more relevant.

In this context, our results revealed significant glomerular changes, with an increased glomerular surface area and Bowman’s space in both STZ-SD and STZ-HPD groups compared to controls. This increase was further amplified in the STZ-HPD group, suggesting that elevated protein intake exacerbates glomerular damage, which is consistent with findings that dietary factors accelerate glomerular injury in DN [5]. In addition to glomerular changes, we observed PTEC hyperplasia in both diabetic groups, with a more pronounced response in the STZ-HPD group. This further suggests that a high-protein diet aggravates tubular injury, reinforcing previous observations that dietary protein overload contributes to tubular stress and dysfunction in DN [7]. Inflammation and oxidative stress play a critical role in the progression of DN. Hyperglycemia activates inflammatory pathways, including cytokine production, contributing to kidney damage. Simultaneously, oxidative stress, characterized by excess reactive oxygen species (ROS) and reduced antioxidant capacity, exacerbates inflammation and fibrosis, accelerating kidney dysfunction [30,31]. In this study, HPD induced significant changes in inflammatory markers, including a notable increase in *Ccl2* expression and a rise in *Tnf* levels compared to the STZ-SD group. These shifts in the inflammatory profile suggest that a HPD exacerbates kidney damage in DN. Overall, these findings demonstrate that a HPD amplifies both inflammation and oxidative stress, two key drivers of DN progression. Surprisingly, while these renal alterations were evident, no significant differences in fibrosis scores were observed between the STZ and STZ-HPD groups, indicating that the high-protein diet did not exacerbate fibrotic processes beyond the effects induced by STZ alone. This suggests that, at this stage of DN, fibrosis is not a predominant feature in this model. The development of renal lesions, such as fibrosis, appears to be model-dependent, with lesion progression varying based on the specific experimental conditions and the duration of dietary intervention. Studies have shown that tubulointerstitial lesions become evident after approximately 14–16 weeks in the Black and Tan BRachyury (BTBR) ob/ob mouse model [32] and in 16 weeks in the 5/6 nephrectomy (5/6 Nx) 129/Sv mouse model [33]. These findings suggest that the 12-week duration of the HPD used in our study may not have been sufficient to induce significant fibrosis, in line with previous studies indicating that longer exposure times are needed to potentially observe fibrotic changes [32,33]. Therefore, the timeline for fibrosis development is dependent on both the model used and the duration of the dietary intervention, highlighting the need for extended experimental periods to fully capture the progression of renal lesions in diabetic nephropathy models.

Like HPDs, we know that high-salt [34] and high-fat diets [35] play an important role in the development of DN. To take this further, it would be interesting to investigate the pathophysiological mechanisms of DN in mice subjected to these various dietary regimens, aiming to compare how they influence the progression of the disease.

Our study has limitations due to the use of the STZ-induced diabetic mouse model. While being widely used to study DN, this model does not fully replicate the complex pathophysiology of human pathology. STZ primarily targets and destroys pancreatic beta cells, leading to hyperglycemia, but it does not mimic the gradual onset and progression of type 2 diabetes, which is more common in humans. Additionally, the genetic background and strain of the mice can significantly influence the susceptibility to DN and the severity of kidney injury, making it challenging to generalize findings across different studies [36]. Another limitation is the variability in the development of key features of advanced human DN, such as nodular glomerulosclerosis and severe tubulointerstitial fibrosis. Many STZ-induced models exhibit only mild to moderate glomerular and tubular changes, which do not fully capture the advanced stages of human DN [37]. In summary, while the STZ-induced diabetic mouse model provides valuable insights into the pathogenesis of DN, its limitations in replicating the full spectrum of human DN highlight the need for more robust and clinically relevant models to advance our understanding and treatment of this complex disease, making our work even more relevant.

In summary, this research, which employed a STZ-induced diabetic C57Bl/6J mouse model, shows that protein intake worsens key indicators of DN compared to the standard STZ model. These results underscore the critical role of dietary factors in the progression of DN and indicate that this model can provide important insights into the underlying mechanisms of the disease. Offering clear benefits over current models, it opens up new avenues for studying CKD and serves as a valuable tool for future investigations.

## 4. Materials and Methods

### 4.1. Animal Experimentation

Official French regulations for the use and care of laboratory animals were followed throughout the experiments. The experimental protocol was approved by the local ethics committee (CE2A, Dijon, France) for animal experimentation (APAFIS no. 39296, approval date: 19 December 2022).

### 4.2. Animals and Diet

Eight-week-old C57Bl/6J male mice from Janvier Labs (Le Genest Saint Isle, France) were housed under a 12-h light/dark cycle and fed a standard diet *ad libitum*. Diabetes was induced via intraperitoneal (IP) injection of streptozotocin (STZ; 45 mg/kg/day) in a sodium citrate solution (0.1 M, pH 4.5) for five consecutive days. Non-diabetic control mice were administered citrate buffer IP for five days. Two weeks following STZ injection, mice with random glucose levels ≥ 230 mg/dL were considered diabetic. At this point, diabetic mice were randomly assigned to either a standard (E15202-24, SSNIFF, Soest, Germany) (STZ-SD mice) or a high protein diet (E15209-34, SSNIFF, Soest, Germany, 45 kcal% protein) (STZ-HPD mice) *ad libitum* with free access to drinking water for 12 weeks. Diets were administered as pellets.

During the last week, mice were single-housed in metabolic cages (Techniplast, 3600M021, Decines-Charpieu, France) for 24 h for urine collection. After deep anesthesia by intraperitoneal injection of ketamine/xylazine (100 mg/kg and 10 mg/kg, respectively), animals were euthanized by exsanguination followed by cervical dislocation to ensure death. Tissues were collected and snap frozen in liquid nitrogen or fixed in 4% paraformaldehyde before histological analysis. Trunk blood was collected to determine endocrine and biochemical parameters.

### 4.3. Biochemical Markers

Tail-vein blood glucose was evaluated using a glucometer (MyLife Glucometer, Pura, Milan, Italy), while urinary glucose was determined using a colorimetric test (Glucose GOD FS; DiaSys Diagnostic Systems; Grabels, France). Insulin and glucagon were assessed from 1 single sample using the Bio-Plex Pro Mouse Diabetes 8-Plex Assay (Biorad Laboratories, Hercules, CA, USA, cat.171-A7001M). Circulating levels of angiotensin II were determined by ELISA (Angiotensin II EIA Kit, Merck, Darmstadt, Germany, #RAB0010). Blood and urine creatinine were measured by the “Lipidomic Analytical Platform” of the University of Burgundy as in [38]. Urinary albumin was measured in the urine pre- and post-treatment using an enzyme-linked immunosorbent assay (ELISA) kit (Mouse Albumin ELISA Kit; Bethyl Laboratories, Montgomery, TX, USA). Urinary levels of KIM-1 (Mouse TIM-1/KIM-1/HAVCR Quantikine ELISA Kit; R&D Systems, Minneapolis, MN, USA; #MKM100) and N-GAL (Mouse NGAL-Neutrophil Gelatinase Associated Lipocalin ELISA Kit; Assay Genie, Dublin, Ireland; #MOES01298) were assessed by ELISA following the manufacturers’ recommendations.

### 4.4. Histology

Histological and immunohistochemical experiments were carried out in the ImaFlow core facility (US58 BioSanD, INSERM, University of Burgundy, Dijon, France). After collection, kidneys were cut longitudinally and directly fixed in 4% formalin (Labelians, Nemours, France, FPC60FT) for 48 h at room temperature (RT) and then dehydrated, impregnated with paraffin (Leica ASP300 automatic dehydration/impregnation machine), and embedded in paraffin (Leica EG1160 embedding station). The paraffin blocks were sectioned (5 µm thickness) using a microtome (Leica HistoCore Autocut R, Nanterre, France). The sections were unfolded and then deposited on coated glass slides and dried overnight in an oven at 37 °C. Tissue sections were deparaffinized in xylene and rehydrated in serial alcohol solutions, respectively. To evaluate the remodeling of the renal matrix, renal sections were stained with Harris Hematoxylin Eosin Y alcoholic, Periodic Acid Schiff (PAS), or Sirius Red using an automated stainer (Leica AutoStainer XL, Nanterre, France).

For immunohistochemical analysis, tissue sections were deparaffinized in xylene and rehydrated in decreasing concentrations of ethanol. After antigen retrieval, tissue sections were first incubated with the designated primary antibody (Table A1) and then incubated for 60 min at room temperature with a fluorescent-labelled secondary antibody. Sections were then dehydrated and mounted in anti-fading aqueous medium. All images were acquired on an AxioImager M2 microscope (Zeiss, Oberkochen, Germany) equipped with an Axiocam (Zeiss) camera using 20× and 40× objectives or on an Axio Imager M2 microscope (Zeiss) equipped with an AxioCam camera using 20× and 40× objectives for fluorescent labelling. Analyses were then performed using either Image J software 1.54g (NIH, Bethesda, MD, USA) or ICY software V2.4.2 (Institut Pasteur and France-BioImaging, Montpellier, France). Fibrotic score was calculated based on percentage of fibrosis surface over the entire field in Sirius red-stained samples. Podocalyxin, LRP2, and SGLT2 images were analyzed using the Image J analysis software 1.54g (National Institutes of Health, Bethesda, MD, USA). Regions of interest (ROIs) were segmented using the otsu dark automatic thresholding method on the channel of interest after gamma enhancement. ROIs were cleaned up by erosion and the total area in µm was extracted using the metadata of each image. The integrated intensity was measured in the combined ROIs on the raw image. Lipocalin-2 puncta were detected using the spot detector [39] module in ICY (Institut Pasteur, France Bioimaging, Montpellier, France). The detection was then followed by extraction of the integrated intensities of the Lipocalin-2 puncta for each image. Both analysis methods were automatized in-house in the form of an ImageJ macro 1.54g or an ICY protocol to limit user intervention.

### 4.5. PCR

Total RNAs from kidneys were extracted using the TRIzol reagent technique. Purity was checked using the N50 Nanophotometer (Implen GmbH, Munich, Germany). Reverse transcription was performed from 1 µg of total RNA using the iScript cDNA synthesis kit (Biorad Laboratories, Hercules, CA, USA, #1708890). qPCR was performed with the Sybr Green Supermix kit (Biorad Laboratories, Hercules, CA, USA, #1708886) and analyzed with the QuantStudio 3 Real Time PCR System (ThermoFisher, Illkirch-Graffenstaden, France). For each gene, a standard curve was generated from four cDNA dilutions (1:5 to 1:100) and used to determine the relative change in gene expression after normalization with the geometric mean of the reference genes *Rpl19*, *Rpl32*, *Rplp0,* and *Hprt*. The nucleotide sequences of the primers used in this project are presented in Table A2.

### 4.6. Statistical Analysis

Statistical analysis was performed with GraphPad Prism (version 10.2.3 for Windows, San Diego, CA, USA) by analysis of variance (ANOVA) followed by Tukey–Kramer post-hoc test for multiple comparisons. Statistical significance was set at *p* < 0.05. All summary results are presented as mean ± standard error of the mean.

## Data Availability

All animal studies were performed and the data were generated at the INSERM U1231 research center. All data and information are available in the main text. Any additional information required to reanalyze the data reported in this paper is available from the lead contact upon request. Data are located in controlled access data storage at Université de Bourgogne.

## Abbreviations

The following abbreviations are used in this manuscript:

ACR	Albumin-to-Creatinine Ratio
AGEs	Advanced Glycation End-products
Ccr	Creatinine Clearance
CKD	Chronic Kidney Disease
DN	Diabetic Nephropathy
GFR	Glomerular Filtration Rate
HPD	High Protein Diet
PTEC	Proximal Tubular Epithelial Cells
ROS	Reactive Oxygen Species
SD	Standard Diet
STZ	Streptozotocin

## Appendix A

**Table A1:** Antibodies used for immune staining.

Protein	Antigen Retrieval	Reference	Dilution	Incubation Time
Lipocalin-2	EDTA pH 9.0	Bio Techne–AF1857 (Minneapolis, MN, USA)	1/100	Overnight, 4 °C
LRP2	EDTA pH 9.0	BiCell Scientific-31012 (San Diego, CA, USA)	1/300	1 h, RT
Podocalyxin	EDTA pH 9.0	R&D Systems-AF1556 (Minneapolis, MN, USA)	1/1000	Overnight, 4 °C
SGLT2	EDTA pH 9.0	BiCell Scientific-20802 (San Diego, CA, USA)	1/500	1 h, RT
WT-1	Citrate pH 6.0	BiCell Scientific-00180 (San Diego, CA, USA)	1/400	Overnight, 4 °C

**Table A2:** Primer sequences.

Gene	Forward	Reverse
*Agtr1a*	AACAGCTTGGTGGTGATCGTC	CATAGCGGTATAGACAGCCCA
*Ccl2*	TTAAAAACCTGGATCGGAACCAA	GCATTAGCTTCAGATTTACGGGT
*Dkk3*	CTCGGGGGTATTTTGCTGTGT	TCCTCCTGAGGGTAGTTGAGA
*Hprt*	AGTCCCAGCGTCGTGATTAG	TTTCCAAATCCTCGGCATAATGA
*Il6*	GAACAACGATGATGCACTTGC	TCCAGGTAGCTATGGTATTCC
*Lcn2*	GCAGGTGGTACGTTGTGGG	CTCTTGTAGCTCATAGATGGTGC
*Nox2*	CCTCTACCAAAACCATTCGGAG	CTGTCCACGTACAATTCGTTCA
*Nox4*	GAAGGGGTTAAACACCTCTGC	ATGCTCTGCTTAAACACAATCCT
*Rpl19*	ATGAGTATGCTCAGGCTACAGA	GCATTGGCGATTTCATTGGTC
*Rpl32*	TTAAGCGAAACTGGCGGAAAC	TTGTTGCTCCCATAACCGATG
*Rplp0*	AGATTCGGGATATGCTGTTGGC	TCGGGTCCTAGACCAGTGTTC
*Tim1*	ACATATCGTGGAATCACAACGAC	GCTACGACGTGGGCTACAG
*Tnf*	CCCTCACACTCAGATCATCTTCT	CCTGGACGTTAAAGGTCGTCA
